# Sturge–Weber Syndrome with Bilateral Port-Wine Stain

**DOI:** 10.1155/2022/2191465

**Published:** 2022-04-15

**Authors:** Bishnu Deep Pathak, Shriya Sharma, Aakriti Adhikari, Nabin Simkhada, Bhuwan Ghimire, Nirjala Aryal

**Affiliations:** ^1^Nepalese Army Institute of Health Sciences, College of Medicine, Kathmandu, Nepal; ^2^Department of Internal Medicine, Nepalese Army Institute of Health Sciences, College of Medicine, Kathmandu, Nepal; ^3^Department of Pediatrics, Nepalese Army Institute of Health Sciences, College of Medicine, Kathmandu, Nepal

## Abstract

Sturge–Weber syndrome is a rare congenital neurocutaneous disorder characterized by dermatological, ophthalmological, and neurological manifestations. It occurs due to abnormal persistence of embryonic vascular plexus. Here, we describe a case of four years seven months female with seizures, developmental delay, intellectual disability, and bilateral port-wine stain diagnosed as type I (classical) Sturge–Weber syndrome. The ophthalmological evaluation was unremarkable. Electroencephalogram showed abnormalities suggestive of a structural lesion in the right cerebral hemisphere. CT scan of the head revealed volume loss of right brain parenchyma with linear, cortical, as well as subcortical calcifications more evident in the right hemisphere. The child should be followed up regularly until adulthood for ophthalmological evaluation, recurrence of seizures, and other manifestations of this disorder.

## 1. Introduction

Sturge–Weber syndrome (SWS), also known as “encephalotrigeminal angiomatosis,” is a rare congenital, sporadically occurring neurocutaneous syndrome with an estimated frequency of about one per 50000 live births [1–4]. According to the Roach scale, it is of three types. Type I (classical) presents as port-wine stain and neurological symptoms, often with glaucoma. Type II manifests with port-wine stain and glaucoma but without neurological lesions. Type III (rarest) is characterized by only leptomeningeal angiomas [3, 5].

It is caused by failure of regression of embryonal vascular plexus resulting in the formation of capillary angiomas over the skin, eyes, leptomeninges, and oral cavity [5–7]. The cutaneous angiomas called “Port-wine stains” or “Nevus flammeus” are typically located over the face along the dermatomes supplied by ophthalmic (V_1_) and maxillary (V_2_) branches of the trigeminal nerve. They are usually unilateral, but sometimes, they may be bilateral or absent or widespread to other parts of the body [2]. Ocular involvement includes the development of glaucoma and retinal/choroidal angiomas [5, 8]. About 40% of the cases may develop oral lesions such as excess gingival growth and asymmetric jaw growth [9]. Intracranial vascular anomaly includes leptomeningeal angiomas, located mostly in the occipital and posterior parietal lobes. Laminar cortical necrosis and calcification may occur secondary to ischemia caused by angiomatosis. Clinically, the patient presents with seizures, recurrent stroke-like episodes, mental retardation, and developmental delays [4, 5].

We report a case of four years and seven-month-old female child with SWS who presented with seizures, developmental delay, bilateral facial port-wine stain, and intracranial calcification.

## 2. Case Presentation

A four years and seven-month-old female child presented to the pediatric outpatient department (OPD) with the chief complaints of loss of consciousness with abnormal body movements three episodes for the last two days. According to the informant (her mother), two days back, initially, she had a jerky movement of the left upper limb which lasted for about one minute, and later, it involved all four limbs. She also had a loss of consciousness that started with abnormal body movements and lasted for about two minutes. The mother noticed frothing at her mouth, stiffening of upper limbs, and loss of bladder control during the episode. The mother also gave a history of delayed developmental milestones like head holding and walking without support after six months and two years of age, respectively. There was no history of fever, headache, neck stiffness, photophobia, visual disturbances, head injury, weakness, and any drug intake. The patient had no childhood disorder as such. Her perinatal period was uneventful, and she had completed immunization as per Nepal Government Expanded Program on Immunization (EPI) schedule.

**Figure 1:**
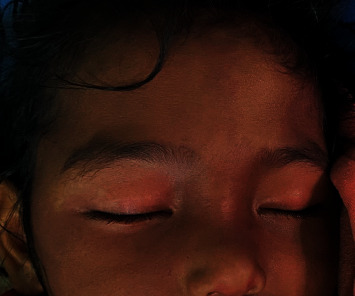
Port-wine stain over the right part of the forehead, periorbital area, and maxillary area (V_1_ and V_2_ dermatome of the right trigeminal nerve) and left part of the forehead and periorbital area (V_1_ dermatome of left trigeminal nerve).

On examination, her vitals were within normal limits. There was no pallor, icterus, lymphadenopathy, clubbing, cyanosis, edema, and dehydration. On general body examination, she had a light pinkish-purple patch over the right forehead, periorbital, and maxillary regions (V_1_ and V_2_ distribution of right trigeminal nerve) and a similar one the over left part of the forehead (V_1_ division of left trigeminal nerve) as shown in Figure 1. There were no signs of meningeal irritation. Neurological, cardiovascular, respiratory, and abdominal examinations showed normal findings.

**Figure 2:**
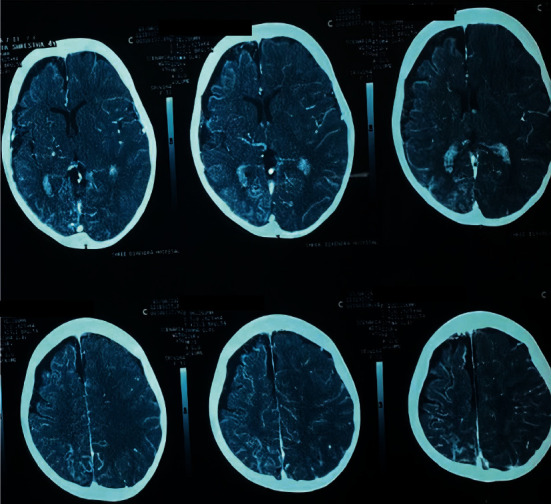
CT head (with angiography) showing volume loss of right brain parenchyma and linear calcifications.

She was put on an oral antiepileptic drug (tablet oxcarbamazepine 11.5 mg/kg/day) and was monitored on regular basis for recurrence of seizures. Baseline investigations were sent which included complete blood count (CBC), random blood sugar (RBS), Renal Function Test (RFT) with serum electrolytes, liver function test (LFT), plain chest X-ray, and electrocardiogram. All of them showed normal results. Then, a sleep electroencephalogram (EEG) was done which showed abnormality suggestive of a structural lesion in the right cerebral hemisphere. Finally, noncontrast computerized tomography (NCCT) head followed by CT-angiography was done as shown in Figure 2. It showed volume loss of right brain parenchyma with linear calcifications in cortical as well as subcortical white matter, more evident in the right hemisphere, hypoplastic right posterior communicating (PCOM) artery, relative paucity of cortical vein on right hemisphere, and prominent right internal cerebral vein with its collateral in the basal ganglia region.

In a nutshell, flat pinkish-purple facial birthmark (port-wine stain), focal and generalized seizures, developmental delay, radiographic findings of white matter calcifications, and vascular abnormalities were suggestive of Sturge–Weber syndrome type I.

On ophthalmological evaluation, her intraocular pressure was normal with no evidence of retinal, choroidal, episcleral, and conjunctival hemangiomas.

## 3. Discussion

By Roach scale, our case is type I SWS because of the presence of port-wine stain and neurological features (seizure and developmental delay). The port-wine stain was limited to the face along the dermatomal division of the trigeminal nerve. In a report of 14 cases by Parisi L. et al., 3 out of 14 cases presented with capillary angiomas to other body parts such as lower limbs, breech, and omphalic areas [7]. Usually, facial port-wine stain is unilateral as reported by Gill NC et al [5]. To the contrary, it is bilateral in our case and that reported by Mukhopadhyay S [9]. In another case report, facial nevus is absent causing a diagnostic dilemma. Such a variant is type III and is the rarest one [3].

In our case, the seizure has started after infancy (that is, at four years of age) which is in contrast to Zansmera P. et al., where the seizure started at nine months of age only [3]. Ocular involvement has not been evidenced till now in our case. However, other studies show ocular manifestations in the form of dilated ocular vessels, glaucoma, and choroid angioma/megalophthalmos/megalocornea [5, 7, 9]. A few studies reported massive gingival overgrowth and bleeding gums [2, 9], which is not present in this case.

SWS is a multisystem disorder that requires the combined efforts of pediatricians, neurologists, ophthalmologists, dermatologists, neurosurgeons, and other healthcare professionals along with good psychosocial support for the patient as well as family members. Regular follow-up is needed [7]. In this context, there is a limitation associated with this case report to be mentioned. We have not been able to follow up on the case due to noncompliance of the patient party despite adequate counseling at the time of discharge from the hospital.

In our case, the child was not brought to the hospital for facial pigmentation and delayed developmental milestones. After she developed seizures multiple times, then she was brought into consultation, and thereby, she was diagnosed with SWS and treated accordingly. From this, we can infer that if any child with a facial birthmark shows significant delays in developmental milestones, it should be taken seriously by the parents and the appropriate medical consultation should be done. The clinician should also suspect SWS in that condition.

## Data Availability

No data were used to support this study.
